# Urban–Rural Disparities in the Magnitude and Determinants of Stunting among Children under Five in Tanzania: Based on Tanzania Demographic and Health Surveys 1991–2016

**DOI:** 10.3390/ijerph18105184

**Published:** 2021-05-13

**Authors:** Wenjun Zhu, Si Zhu, Bruno F. Sunguya, Jiayan Huang

**Affiliations:** 1 School of Public Health, Fudan University, Shanghai 200433, China; wjzhu20@fudan.edu.cn (W.Z.); zhus17@fudan.edu.cn (S.Z.); 2 Key Lab of Health Technology Assessment, National Health Commission of the People’s Republic of China, Shanghai 200433, China; 3 Global Health Institution, Fudan University, Shanghai 200433, China; 4 School of Public Health and Social Sciences, Muhimbili University of Health and Allied Sciences, Dar es Salaam P.O. BOX 65001, Tanzania; sunguya@gmail.com

**Keywords:** stunting, Demographic and Health Surveys, under-5 children, malnutrition, determinant, interaction

## Abstract

Our study aims to examine the disparity of under-5 child stunting prevalence between urban and rural areas of Tanzania in the past three decades, and to explore factors affecting the rural–urban disparity. Secondary analyses of Tanzania Demographic and Health Surveys (TDHS) data drawn from 1991–1992, 1996, 1999, 2004–2005, 2009–2010, and 2015–2016 surveys were conducted. Under-5 child stunting prevalence was calculated separately for rural and urban children and its decline trends were examined by chi-square tests. Descriptive analyses were used to present the individual-level, household-level, and societal-level characteristics of children, while multivariable logistic regression analyses were performed to examine determinants of stunting in rural and urban areas, respectively. Additive interaction effects were estimated between residence and other covariates. The results showed that total stunting prevalence was declining in Tanzania, but urban–rural disparity has widened since the decline was slower in the rural area. No interaction effect existed between residence and other determinants, and the urban–rural disparity was mainly caused by the discrepancy of the individual-level and household-level factors between rural and urban households. As various types of determinants exist, multisector nutritional intervention strategies are required to address the child stunting problem. Meanwhile, the intervention should focus on targeting vulnerable children, rather than implementing different policies in rural and urban areas.

## 1. Introduction

Achieving and maintaining balanced nutrition is a critical challenge for global health, and improving child nutrition is one of the key components. Since the first 5 years of life is the peak period of growth and development, an under-5 child’s malnutrition is strongly associated with severe dysfunction, mental retardation, and poor ability to work [1], which would impose a heavy economic burden on society [2].

Key anthropometric indicators of nutritional status include stunting, underweight, and wasting [3]. Stunting is the manifestation of chronic undernutrition, while underweight and wasting reflect acute nutritional distress [4]. Compared to the latter two indicators, stunting may leave children with lifelong, possibly irrevocable, consequences. Reduction in global stunting is one of the critical indicators within Sustainable Development Goals (SDGs), and the World Health Organization (WHO) set the target of achieving a 40% reduction in stunting by 2025 [5,6].

About 80% of children with stunting came from 14 countries, one of which is Tanzania [7]. According to the 2015–2016 Tanzania Demographic and Health Survey and Malaria Indicator Survey, one in three children under 5 years old in Tanzania was stunted [8]. In the meantime, the global level of this indicator was 22.9% [9], demonstrating that the stunting prevalence of Tanzania was about 50% higher than the global average. Moreover, the burden of stunting varied widely between regions and across sociodemographic divides in Tanzania [10,11]. Similar to inequity in the nutritional landscape elsewhere, children in the rural area bore the biggest brunt of the burden [11,12,13].

Previous studies showed that the inequity between rural and urban areas was associated with several factors, including access to public services, education, and wealth [14,15]. Their findings also showed that understanding determinants of stunting in each context can help to implement interventions for urban and rural areas separately. As far as we know, such kind of evidence from Tanzania is limited. The existing ones are commonly based on either a small sample size targeting specific regions or few factors without the newest data [16,17,18]. Therefore, the primary purpose of the study is to examine the trends in under-5 child stunting prevalence between the rural and urban areas in Tanzania during the past three decades. The second purpose is to identify the factors associated with stunting and to find out whether these factors played different roles in rural and urban stunting prevalence.

## 2. Materials and Methods

### 2.1. Data Sources

The data were extracted from the six rounds of the Tanzania Demographic Health Surveys (TDHS) which were conducted in 1991–1992, 1996, 1999, 2004–2005, 2009–2010, and 2015–2016. TDHS, which is a part of the worldwide DHS program, is carried out by the Tanzania National Bureau of Statistics and other government personnel every five years in all regions of the country [8]. The surveys mainly use two types of tools: biomarker testing and questionnaires. Well-trained field staff were responsible for data collection. All tests were standardized by DHS to guarantee the continuity and comparability of the data through different surveys. In our analysis, the height and weight measurements of children came from biomarker testing, and the demographic characteristics of children, along with household and societal characteristics, came from questionnaires.

### 2.2. Study Population and Sample Size

The detailed TDHS sampling methodology has been published previously [8]. In brief, TDHSs were cross-sectional in design and were representative at both national and regional levels. The first three rounds of the TDHS, which were conducted in 1991–1992, 1996, and 1999, used three-stage sampling: wards/branches were selected at the first stage, enumeration areas (EAs) at the second stage, and households at the third stage. The sample design for 2004–2005, 2009–2010, and 2015–2016 TDHSs were done in two stages: EAs were selected at the first stage and households at the second stage. EAs were delineated according to the latest Tanzania Population and Housing Census. All children aged under 5 living in the sample households were included in the surveys. The overall sample size of under-5 children was 41,297, with 7287, 6080, 2839, 7852, 7526, and 9713 in each round. Due to the focused research objectives and less investment, the sample size of 1999 TDHS was small [19].

### 2.3. Measurement of the Outcome Variable

The primary outcome was stunting prevalence. The WHO Child Growth Standards in 2006 was referred to when determining the child’s nutritional status [20]. Stunting prevalence was defined as the percentage of children aged 0 to 59 months whose length/height-for-age Z score is below minus two standard deviations from the median of 2006 WHO Child Growth Standards [7,20,21]. Based on the child’s sex, age, and length (if aged <2 years) or height (if aged ≥2 years), SPSS macro provided by the WHO (http://www.who.int/childgrowth/software/en/) was applied to calculate the Z scores for each child. Children were excluded if they lacked anthropometric measures data which are required for calculating Z score. Children with implausible height values (length/height-for-age Z score below −6 or above +6) were also excluded. Therefore, final samples of children under five in the present study were 37,409 in total and were distributed as follows: 6587 (1991–1992), 5437 (1996), 2556 (1999), 7231 (2004–2005), 6597 (2009–2010), and 9001 (2015–2016).

### 2.4. Independent Variables

The independent variables were selected according to the results of the literature review and the availability of the data sources [1,22,23,24,25,26,27,28]. These selected variables were classified into three categories according to the UNICEF conceptual framework of malnutrition causation: immediate (individual-level), underlying (household-level), and basic (societal-level) determinants [29].

Individual-level determinants consisted of child characteristics, including age, sex, birth weight, the month of breastfeeding, and place of delivery. Household-level determinants mainly referred to the characteristics of the child’s mother, including the age at her first birth, the total number of children she ever gave birth to, her body mass index (BMI), current marital status, highest education level, and occupation. Besides these, the sex of the household head, source of drinking water, and types of their toilet were also considered. Societal-level determinants included the rural/urban residence of households.

The cutoff point of low birth weight was 2500 g. Mothers with BMI <18.5 kg/m^2^ were defined as underweight, ≥25 kg/m^2^ as overweight, and ≥30 kg/m^2^ as obese. The classification of urban or rural residence was based on the location of sampled clusters [7].

### 2.5. Statistical Analysis

SPSS 22.0 (IBM, Armonk, NY, USA) was used to administer and analyze the data. All samples were weighted according to the “sample weights” variable generated by the TDHS. Otherwise, the standard errors would be underestimated due to the stratified two-stage cluster design of TDHS. It can also help to generalize the findings to the entire country. The stunting prevalence of rural and urban under-5 children in six rounds of TDHS was calculated separately, while the chi-square tests were applied to assess the trends. Descriptive analyses were presented to describe the characteristics of rural and urban children using the latest TDHS (2015–2016). Meanwhile, the chi-square test was performed to compare the differences between rural and urban groups.

Based on six rounds of TDHS, we separated the datasets according to the residence, and two multivariable logistic regression models were conducted to explore how individual-level and household-level factors were associated with urban/rural childhood stunting. The dependent variables were urban/rural under-5 child stunting prevalence, and the independent variables were individual-level and household-level determinants. Since mother’s BMI was not recorded in the 1999 TDHS, this period of sample was not included in the logistic regressions. In addition, the backward stepwise method was used to select variables.

To explore whether the risk of covariates on stunting was modified by rural/urban residence, interactions between residence and other individual-level and household-level covariates were estimated using the latest TDHS (2015–2016). Several new multivariable logistic regression models were constructed. In each model, we included an interaction term associated with the residence and one of the covariates, while setting other covariates as confounders. The dependent variable was the total under-5 child stunting prevalence. Relative excess risk due to interaction (RERI) was calculated to estimate interaction on an additive scale, as it more appropriately reflected the biological interaction [30]. If biological interaction does not exist, RERI is equal to 0. The confidence interval (CI) of RERI was estimated based on methods proposed by Andersson and Knol [31,32]. A *p*-value < 0.05 was considered significant.

## 3. Results

### 3.1. Trends in the Prevalence of Stunting in Urban and Rural Areas

**Figure 1:**
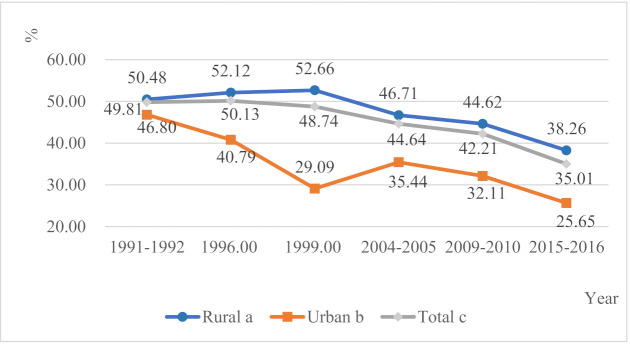
Trends of the prevalence of stunting in the rural and urban areas in Tanzania during 1991–2016; Tanzania Demographic and Health Surveys. a. *p* for trend <0.001, χ^2^ for trend = 290.39; b. *p* for trend <0.001, χ^2^ for trend = 167.58; c. *p* for trend <0.001, χ^2^ for trend = 481.63.

Although overall stunting prevalence in under-5 Tanzania children had decreased significantly (*p* < 0.001) in the past three decades, the burden of stunting among children in the rural population was persistently high (Figure 1, Table S1). In 1991–1992, stunting prevalence was 50.48% (95% CI: 49.15–51.80%) in rural area and 46.80% (95% CI: 43.98–49.63%) in urban area. By 2015–2016, stunting prevalence dropped to 38.26% (95% CI: 37.08–39.43%) in rural area and 25.65% (95% CI: 23.85–27.45%) in urban area. While the general compound annual reduction rate of stunting was 6.81%, the rate of decline was higher among urban area (11.33%) compared to that of rural area (5.39%). Such differential decline has led to a rate difference of 12% between urban and rural areas in 2016 compared to less than 4% in 1991–1992.

### 3.2. Characteristics of Urban and Rural Children

Individual-level and household-level characteristics of under-5 children among urban and rural areas in 2015–2016 TDHS are presented in Table 1. Considering the individual-level factors, the great majority (88.31%) of urban children were born in medical institutions. However, in the rural area, this proportion was only 54.42%, and the rest of them were born at home. Considering household-level factors, compared with urban households, the mothers from rural tended to give birth at an earlier age, have more children and lower education levels, and engage in agricultural works. Mothers being overweight or obese were of great concern in the urban area (40.20%), but not in rural (18.82%). Besides, sanitation problems were faced by a large number of rural households, since most of them used pit latrine (76.73%) instead of the flush toilet (2.74%). The sanitation condition was much better in the urban area.

### 3.3. Determinants of Child Stunting in the Urban and Rural Areas

Several factors were associated with child stunting in the urban area. In the individual-level, there was lower stunting odds among children who were younger, female (odds ratio (OR): 0.63, 95% CI: 0.55–0.72, *p* < 0.001), born in medical institutions (OR: 0.61, 95% CI: 0.45–0.83, *p* = 0.002), had normal or high birth weight (OR: 0.46, 95% CI: 0.39–0.55, *p* < 0.001), and had a shorter duration of breastfeeding. At the household-level, the odds of stunting were lower in children whose mother had fewer children, was obese, and attended a higher level of education. Besides, compared with non-working mothers, children with farmer mothers had higher odds of stunting (OR: 1.34, 95% CI: 1.12–1.60, *p* = 0.003). Additionally, children born in households whose heads were male had lower chances of stunting (OR: 0.65, 95% CI: 0.55–0.76, *p* < 0.001), and the type of toilet was significantly associated with childhood stunting, as children from the family who had worsened sanitation (pit latrine, no facility/bush/field) were more likely to become stunted (Table 2).

In the rural area, except for the total number of children, the highest education level of the mother, and the source of drinking water, the determinants of child stunting were similar to urban children (Table 3). Particularly, the odds of stunting in children aged 24–35 months was 3.53 times higher than the odds in children aged 0–11 months. Compared to underweight mothers, overweight and obese mothers halved the odds of child stunting. Besides, children whose family used pit latrine were associated with a 129% increase in the odds of stunting compared to children whose family used the flush toilet. These three predictors showed a greater influence on rural children than on urban children.

In addition, logistic regressions were applied to find out whether interaction effects existed between residence and other individual-level and household-level covariates. The results showed that no statistically significant interaction was found between residence and other covariates (Table 4).

## 4. Discussion

Our findings showed that stunting prevalence in the urban Tanzanian area decreased significantly and has met the target 5 years in advance—reducing the prevalence of stunting to 28% by 2021 [33]. However, given the present trend, the decline of stunting prevalence in the rural area is still a challenge to the same target. Moreover, stunting gaps between urban and rural under-5 children widened during the past three decades, which underscored the need to identify and address the causes of child stunting, especially in the rural Tanzanian area. In accordance with the current results, previous studies also found that nutritional disparities between urban and rural children were presented in almost all low- and middle-income countries [34,35,36,37].

In terms of individual-level determinants, five predictors are associated with under-5 child stunting prevalence in both urban and rural areas. Elder children had a higher possibility of being stunted, and this may be correlated with the fact that children who received a longer duration of breastfeeding also tended to be stunted. A possible explanation for this might be that prolonged breastfeeding delayed the complementary food intake of children [38]. Present recommendations are that babies should be put on the breast within 1 h after birth, be exclusively breastfed for the first 6 months, and be breastfed along with complementary foods for an additional 18 months or longer [2,39,40]. Besides, in contrast to elder urban children, elder rural children were more vulnerable since their odds of stunting were almost double, or even triple, the odds in children aged 0–11 months. These differences can be explained in part by the fact that most of the rural children are fed by homegrown products while their urban counterparts are exposed to a variety of commercial baby foods which could provide children with necessary nutrients [41]. Furthermore, rural children are often exposed to either lack of dietary diversity or a shortage of food in the dry seasons [42]. Therefore, it is important to promote the quantity and quality of complementary foods in the rural area to improve feeding practices and thus the nutritional status of children.

Several household-level determinants were also associated with child stunting in urban or rural areas. Consistent with previous studies, the results from the present study revealed that a higher level of maternal education is associated with a lower risk of child stunting in urban areas [17,38]. Maternal education impacts child nutrition partly through diets and the use of healthcare and ante/post-natal facilities [36,43]. Educated mothers know how to feed their children correctly [44,45]. Besides, the occupation of the mother, which is highly associated with her education level, also had a significant effect on child stunting in both rural and urban areas. On the one hand, the stunting risk of the child having an agricultural-worker mother was the highest among all the children. Agricultural activities can take a large quantity of time for females, reducing the care and attention they give to their children. A previous study also discovered that interventions to alleviate the negative effects of mothers’ working status may contribute to reducing rural–urban children’s nutritional disparities [36]. On the other hand, increasing mother’s labor supply will increase the investment for children’s nutrition [46,47], and this may explain why rural housewives who do not earn an income had a negative impact on child nutrition. To address this problem, more attention and resources should be paid and allocated to the agricultural and no-job mothers as well as their children’s nutritional status.

Moreover, children from the families using pit latrine or not even having a toilet had a higher risk of stunting compared to others, especially in the rural area. Poor household sanitation has also been found to be an important driver of stunting in many other low- and middle-income countries [48,49]. Using pit latrine or defecating in the open may promote the spread of disease caused by fecal–oral transmission, including diarrhea, typhoid, hepatitis, and so on. According to the WHO, such kind of disease is a leading cause of malnutrition in under-5 children, as it reduces the absorption of sufficient nutrients [50]. As there were still 97% of rural children and 60% of urban children unable to access flush toilets in 2015–2016, poor household sanitation should be targeted to improve under-5 children’s health and nutritional status alongside nutritional complement programs in Tanzania [51].

Additionally, the results of the interaction examination revealed that there was no interaction between residence and any individual-level or household-level determinants. This may be because the residence was not a direct cause of child stunting, and the rural–urban disparity in child stunting was mainly caused by the socioeconomic discrepancy between rural and urban households. Therefore, there is no need to issue different nutritional interventions towards rural and urban households [35]. The intervention programs should rather focus on targeting vulnerable groups and implementing nutritional or sanitation improvement programs in both areas. Besides, the various types of stunting determinants indicated that child malnutrition is not only a health issue but a comprehensive one. It requires the health sector to work with the financial, agricultural, and educational sectors [52,53]. Thus, multisectoral nutritional intervention strategies are needed to improve the nutrition and growth of under-5 children in Tanzania.

To our knowledge, this study is the first to examine long-term trends in stunting among under-5 children in Tanzania by rural–urban setting. Logistic regressions were performed to find out determinants of child stunting in urban and rural areas respectively, and interaction effects were examined between residence and other covariates to test whether different policies were needed in urban or rural areas. However, evidence from this study should be interpreted carefully, owing to the following limitations. Firstly, studies using online open datasets are limited in the selection of variables since the variables of interest may not be found in the datasets. Based on our results, further studies can explore more influential factors on child stunting by primary data gathering. Another limitation is recall bias, as much information about under-5 children was collected through their mothers’ memories, such as birth weight and month of breastfeeding, etc. Thirdly, the 1999 stunting prevalence of urban children was visibly lower than expected. This may be due to the small and unrepresentative sample size of 1999 TDHS. We also excluded this part of data from logistic regressions because of incomplete variables, and this may introduce bias to trends examination and determinants exploration. Despite these limitations, our findings are still valuable because of the insight provided into the rural–urban differential in child stunting.

## 5. Conclusions

In conclusion, although the stunting prevalence of under-5 children has declined in the past three decades in Tanzania, the nutritional disparity between urban and rural children has widened, and stunting is still an overwhelming phenomenon in the rural area. Since there was no interaction between residence and other influential factors on child stunting, the nutritional disparity was mainly attributed to the socioeconomic imbalance between rural and urban households. Therefore, anti-malnutrition initiatives should be directed at vulnerable children, such as children from agricultural families, and when the gaps between rural and urban in the economy, education, food supply, and women’s status gradually narrow, the gap in child nutrition will also vanish.

## Figures and Tables

**Table 1:** Individual-level and household-level characteristics of under-5 children among rural and urban areas based on 2015–2016 Tanzania Demographic and Health Surveys (weighted frequency) (*n* (%)).

Variables	Urban (*n* = 2284)	Rural (*n* = 6531)	χ^2^	*p*
Individual-level				
Age of child				
0–11	520 (22.77)	1450 (22.20)	24.65	0.04
12–23	573 (25.09)	1481 (22.68)		
24–35	424 (18.56)	1264 (19.35)		
36–47	411 (17.99)	1169 (17.90)		
48–59	355 (15.54)	1166 (17.85)		
Missing	1 (0.04)	1 (0.02)		
Sex of child				
Male	1179 (51.62)	3291 (50.39)	1.96	0.31
Female	1105 (48.38)	3240 (49.61)		
Birth weight				
Low	277 (12.13)	432 (6.61)	2.16	0.34
Normal or high	1760 (77.06)	3099 (47.45)		
Missing	247 (10.81)	3000 (45.93)		
Month of breastfeeding				
<6	263 (11.51)	699 (10.70)	3.6	0.06
6–12	280 (12.26)	834 (12.77)		
13–24	336 (14.71)	945 (14.47)		
>24	22 (0.96)	89 (1.36)		
Never breastfed	29 (1.27)	39 (0.60)		
Missing	1354 (59.28)	3925 (60.10)		
Place of delivery				
Home	267 (11.69)	2977 (45.58)	861.88	<0.001
Medical institutions	2017 (88.31)	3554 (54.42)		
Household-level				
Mother’s age at first birth (Y)				
0–14	31 (1.36)	189 (2.89)	246.99	<0.001
15–19	1149 (50.31)	4083 (62.52)		
20–24	807 (35.33)	1935 (29.63)		
≥25	297 (13.00)	324 (4.96)		
Total number of children ever born				
1–2	1154 (50.53)	2060 (31.54)	419.44	<0.001
3–4	707 (30.95)	1873 (28.68)		
≥5	423 (18.52)	2598 (39.78)		
Mother’s body mass index				
Underweight	126 (5.52)	485 (7.43)	690.05	<0.001
Normal	1240 (54.29)	4817 (73.76)		
Overweight	501 (21.94)	970 (14.85)		
Obese	417 (18.26)	259 (3.97)		
Mother’s current marital status				
Married	1444 (63.22)	4090 (62.62)	2.49	0.35
Living together	464 (20.32)	1398 (21.41)		
Widowed/divorced/live apart	247 (10.81)	725 (11.10)		
Never married	129 (5.65)	317 (4.85)		
Missing	0 (0.00)	1 (0.02)		
Mother’s highest education level				
No education	208 (9.11)	1683 (25.77)	880.1	<0.001
Primary	1389 (60.81)	4297 (65.79)		
Secondary	625 (27.36)	536 (8.21)		
Higher	62 (2.71)	15 (0.23)		
Mother’s occupation				
Non-agricultural worker	1296 (56.74)	992 (15.19)	2547.8	<0.001
Agricultural worker	351 (15.37)	4755 (72.81)		
Not working	637 (27.89)	783 (11.99)		
Missing	0 (0.00)	1 (0.02)		
Sex of household head				
Male	1817 (79.55)	5531 (84.69)	40.57	<0.001
Female	467 (20.45)	1000 (15.31)		
Source of drinking water				
Piped water	776 (33.98)	2383 (36.49)	34.24	<0.001
Well/spring	658 (28.81)	1568 (24.01)		
Open source water	725 (31.74)	2240 (34.30)		
Others	125 (5.47)	340 (5.21)		
Type of toilet				
Flush toilet	887 (38.84)	179 (2.74)	2301.57	<0.001
Pit latrine	1231 (53.90)	5011 (76.73)		
No facility/bush/field	79 (3.46)	1056 (16.17)		
Others	86 (3.77)	284 (4.35)		
Missing	1 (0.04)	1 (0.02)		

Y, year.

**Table 2:** The determinants of under-5 child stunting in the urban area based on 1991–2016 Tanzania Demographic and Health Surveys.

Variables	Reference	B	SE	Wald χ^2^	OR (95% CI)	*p*
Phase *	1991–1992			43.85	1	<0.001
	1996	−0.32	0.10	9.57	0.73 (0.59,0.89)	
	2004–2005	−0.49	0.10	25.98	0.61 (0.51,0.74)	
	2009–2010	−0.43	0.10	17.48	0.65 (0.54,0.80)	
	2015–2016	−0.74	0.13	33.38	0.48 (0.37,0.61)	
Individual-level						
Age of child (M)	0–11			37.89	1	<0.001
	12–23	0.60	0.15	16.69	1.82 (1.36,2.42)	
	24–35	0.71	0.16	20.48	2.04 (1.50,2.77)	
	36–47	0.50	0.16	9.76	1.65 (1.20,2.25)	
	48–59	0.18	0.16	1.25	1.20 (0.87,1.66)	
Sex of child	Male				1	<0.001
	Female	−0.46	0.07	49.10	0.63 (0.55,0.72)	
Birth weight	Low				1	<0.001
	Normal or high	−0.77	0.09	79.19	0.46 (0.39,0.55)	
Place of delivery	Home				1	0.002
	Medical institutions	−0.49	0.16	9.65	0.61 (0.45,0.83)	
Month of breastfeeding	<6			29.20	1	<0.001
	6–12	0.56	0.14	16.83	1.75 (1.34,2.28)	
	13–24	0.69	0.17	16.16	2.00 (1.43,2.80)	
	>24	0.97	0.21	21.73	2.65 (1.76,3.99)	
	Never breastfed	1.28	0.34	14.23	3.60 (1.85,7.00)	
Household-level						
Mother’s total number of children	≥5			7.90	1	0.019
	3–4	−0.22	0.09	5.75	0.80 (0.67,0.96)	
	1–2	−0.03	0.09	0.15	0.97 (0.81,1.15)	
Mother’s BMI	Underweight			19.54	1	<0.001
	Normal	0.08	0.13	0.41	1.09 (0.84,1.40)	
	Overweight	−0.17	0.15	1.41	0.84 (0.63,1.12)	
	Obese	−0.40	0.17	5.40	0.67 (0.47,0.94)	
Mother’s highest education level	No education			27.48	1	<0.001
	Primary	−0.27	0.11	5.97	0.76 (0.61,0.95)	
	Secondary	−0.74	0.15	24.55	0.48 (0.36,0.64)	
	Higher	−0.79	0.34	5.47	0.46 (0.24,0.88)	
Mother’s occupation	Not working			14.01	1	0.003
	Non-agricultural worker	−0.02	0.22	0.01	0.98 (0.64,1.50)	
	Agricultural worker	0.29	0.09	10.26	1.34 (1.12,1.60)	
	Others	−0.01	0.08	0.02	0.99 (0.85,1.16)	
Sex of household head	Female				1	<0.001
	Male	−0.43	0.08	26.69	0.65 (0.55,0.76)	
Source of drinking water	Piped water			9.16	1	0.027
	Open source water	−0.07	0.08	0.76	0.93 (0.79,1.10)	
	Well/Spring	−0.23	0.08	8.12	0.80 (0.68,0.93)	
	Others	−0.27	0.19	1.88	0.77 (0.52,1.12)	
Type of toilet	Flush toilet			18.51	1	<0.001
	Pit latrine	0.35	0.11	10.43	1.41 (1.15,1.75)	
	No facility/bush/field	0.35	0.29	1.52	1.42 (0.81,2.50)	
	Others	−0.33	0.23	2.03	0.72 (0.46,1.13)	
Constant		2.24	0.44	26.35		

* 1999 Tanzania Demographic Health Surveys (TDHS) did not investigate mother’s BMI, thus this part of sample was not included in the logistic regression. B, coefficient; OR, odds ratio; CI, confidence interval; M, month; BMI, body mass index; SE, standard error.

**Table 3:** The determinants of under-5 child stunting in the rural area based on 1991–2016 Tanzania Demographic and Health Surveys.

Variables	Reference	B	SE	Wald χ^2^	OR (95% CI)	*p*
Phase *	1991–1992			44.78	1	<0.001
	1996	0.01	0.07	0.01	1.01 (0.88,1.15)	
	2004–2005	−0.19	0.07	8.38	0.83 (0.73,0.94)	
	2009–2010	−0.22	0.06	11.41	0.80 (0.71,0.91)	
	2015–2016	−0.48	0.08	34.68	0.62 (0.53,0.72)	
Individual-level						
Age of child (M)	0–11			179.07	1	<0.001
	12–23	0.93	0.10	90.59	2.53 (2.09,3.06)	
	24–35	1.26	0.11	142.75	3.53 (2.87,4.35)	
	36–47	1.04	0.11	95.46	2.83 (2.29,3.48)	
	48–59	0.65	0.11	37.36	1.92 (1.56,2.37)	
Sex of child	Male				1	<0.001
	Female	−0.33	0.04	58.60	0.72 (0.66,0.78)	
Birth weight	Low				1	<0.001
	Normal or high	−0.60	0.06	102.19	0.55 (0.49,0.62)	
Place of delivery	Home				1	0.001
	Medical institutions	−0.21	0.06	10.57	0.81 (0.71,0.92)	
Month of breastfeeding	<6			33.42	1	<0.001
	6–12	0.28	0.09	9.87	1.32 (1.11,1.57)	
	13–24	0.36	0.11	10.15	1.44 (1.15,1.80)	
	>24	0.71	0.13	28.55	2.03 (1.56,2.63)	
	Never breastfed	0.31	0.21	2.19	1.36 (0.90,2.06)	
Household-level						
Mother’s BMI	Underweight			69.62	1	<0.001
	Normal	−0.30	0.08	13.74	0.74 (0.64,0.87)	
	Overweight	−0.70	0.10	49.78	0.50 (0.41,0.60)	
	Obese	−0.89	0.16	30.52	0.41 (0.30,0.56)	
Mother’s occupation	Not working			19.32	1	<0.001
	Non-agricultural worker	−0.47	0.20	5.49	0.62 (0.42,0.93)	
	Agricultural worker	0.12	0.06	4.03	1.13 (1.00,1.27)	
	Others	−0.10	0.08	1.53	0.90 (0.77,1.06)	
Sex of household head	Female				1	0.016
	Male	−0.14	0.06	5.84	0.87 (0.78,0.97)	
Type of toilet	Flush toilet			19.66	1	<0.001
	Pit latrine	0.83	0.20	16.61	2.29 (1.54,3.40)	
	No facility/bush/field	0.76	0.21	12.95	2.13 (1.41,3.22)	
	Others	0.61	0.24	6.46	1.84 (1.15,2.94)	
Constant		0.41	0.30	1.87		

* 1999 Tanzania Demographic Health Surveys (TDHS) did not investigate mother’s BMI, thus this part of sample was not included in the logistic regression. B, coefficient; OR, odds ratio; CI, confidence interval; M, month; BMI, body mass index; SE, standard error.

**Table 4:** The interaction effects between residence and other individual-level and household-level covariates based on 2015–2016 Tanzania Demographic and Health Surveys.

Variables	Categories	Urban		Rural		RERI (95% CI)	p *
		N with/without Stunting	OR (95% CI)	N with/without Stunting	OR (95% CI)		
Sex	Female	266/839	1	1119/2121	1.71 (1.16,2.52)		
	Male	308/870	1.32 (0.84,2.08)	1343/1947	2.19 (1.48,3.24)	0.16 (−1.08,1.40)	0.798
Birth weight	Normal or high	471/1536	1	2256/3843	1.60 (1.20,2.14)		
	Low	104/173	1.67 (0.85,3.27)	206/225	4.11 (2.59,6.53)	1.84 (−0.41,4.10)	0.109
Place of delivery	Medical institutions	484/1516	1	1239/2237	1.76 (1.30,2.37)		
	Home	84/181	1.34 (0.68,2.63)	1172/1739	1.87 (1.34,2.60)	−0.23 (−1.45,0.99)	0.711
Mother’s age at first birth (Y)	≥25	41/256	1	137/188	3.53 (1.57,7.92)		
	20–24	208/599	1.80 (0.84,3.86)	740/1195	2.97 (1.43,6.17)	−1.36 (−5.19,2.48)	0.489
	15–19	316/833	1.38 (0.65,2.93)	1505/2577	2.03 (0.98,4.20)	−1.88 (−5.25,1.50)	0.276
	0–14	9/22	1.09 (0.15,7.81)	80/109	3.39 (1.34,8.59)	−0.23 (−4.99,4.53)	0.925
Total number of children ever born	1–2	287/867	1	776/1284	1.51 (1.05,2.19)		
	3–4	157/550	0.65 (0.37,1.15)	685/1188	1.37 (0.91,2.06)	0.21 (−0.67,1.08)	0.644
	≥5	130/292	0.96 (0.51,1.81)	1001/1597	1.53 (1.03,2.27)	0.05 (−0.98,1.07)	0.928
Mother’s current marital status	Married	355/1088	1	1550/2541	1.77 (1.27,2.47)		
	Living together	128/337	1.36 (0.76,2.44)	493/905	1.65 (1.13,2.42)	−0.48 (−1.65,0.69)	0.425
	Widowed/divorced/live apart	59/187	0.67 (0.31,1.45)	295/430	1.65 (1.07,2.56)	0.21 (−0.85,1.28)	0.693
	Never married	32/97	1.09 (0.42,2.84)	125/193	2.25 (1.21,4.18)	0.39 (−1.45,2.23)	0.679
Mother’s occupation	Non-agricultural worker	305/991	1	322/670	1.49 (0.96,2.32)		
	Agricultural worker	121/230	0.78 (0.43,1.43)	1857/2898	1.57 (1.06,2.31)	0.30 (−0.71,1.31)	0.563
	Not working	148/488	0.87 (0.52,1.45)	282/501	1.51 (0.96,2.37)	0.15 (−0.90,1.20)	0.780
Sex of household head	Male	426/1391	1	2063/3467	1.86 (1.36,2.53)		
	Female	148/319	1.55 (0.90,2.64)	398/601	1.87 (1.28,2.74)	−0.54 (−1.77,0.70)	0.396
Source of drinking water	Others	27/99	1	120/220	2.03 (0.67,6.13)		
	Open source water	177/548	1.81 (0.63,5.14)	850/1390	2.86 (1.06,7.74)	0.03 (−4.06,4.11)	0.990
	Well/spring	183/475	1.64 (0.57,4.70)	568/1000	3.14 (1.16,8.51)	0.47 (−3.75,4.69)	0.828
	Piped water	187/588	1.79 (0.64,5.04)	924/1459	2.80 (1.04,7.57)	−0.03 (−4.05,4.00)	0.999
Type of toilet	Flush toilet	180/707	1	45/134	0.93 (0.44,1.95)		
	No facility/bush/field	28/52	1.10 (0.19,6.47)	390/666	1.98 (1.24,3.16)	0.94 (−1.33,3.21)	0.416
	Pit latrine	343/888	1.17 (0.73,1.87)	1933/3078	2.00 (1.34,2.98)	0.90 (−0.30,2.09)	0.141
	Others	23/63	0.94 (0.30,3.00)	94/191	1.08 (0.58,2.03)	0.21 (−1.25,1.67)	0.781
Age of Child (M)	0–11	67/454	1	258/1192	1.40 (0.94,2.10)		
	12–23	160/413	1.49 (0.88,2.53)	626/855	3.04 (1.89,4.89)	1.15 (−0.59,2.89)	0.195
	24–35	145/279	10.24 (2.56,40.88)	629/635	3.59 (1.73,7.46)	−7.05 (−21.48,7.38)	0.338
	36–47	127/285	0.48 (0.04,5.38)	521/648	1.43 (0.43,4.74)	0.54 (−1.60,2.69)	0.619
	48–59	75/280	0.70 (0.10,5.22)	427/739	1.71 (0.48,6.03)	0.60 (−2.04,3.23)	0.658
Month of breastfeeding	<6	25/238	1	102/597	1.62 (0.91,2.85)		
	6–12	47/233	2.17 (1.12,4.20)	170/664	2.49 (1.43,4.34)	−0.30 (−2.49,1.90)	0.791
	13–24	83/254	3.20 (1.64,6.25)	404/541	6.82 (3.70,12.58)	3.00 (−1.78,7.78)	0.218
	>24	11/11	6.76 (1.43,32.05)	46/43	8.67 (3.42,21.94)	1.29 (−11.99,14.57)	0.849
	Never breastfed	8/15	4.34 (1.07,17.68)	13/22	3.95 (1.35,11.53)	−1.01 (−8.49,6.47)	0.792
Mother’s body mass index	Obese	69/348	1	66/193	1.10 (0.47,2.56)		
	Overweight	115/386	0.78 (0.36,1.70)	333/636	1.59 (0.81,3.11)	0.70 (−0.84,2.24)	0.371
	Normal	348/886	1.22 (0.64,2.33)	1857/2948	2.07 (1.13,3.80)	0.75 (−1.00,2.50)	0.401
	Underweight	41/84	1.47 (0.50,4.32)	204/282	2.30 (1.17,4.52)	0.73 (−1.68,3.14)	0.553
Mother’s highest education level	Higher	6/56	1	1/13	0.12 (0.01,1.52)		
	Secondary	119/506	0.75 (0.19,2.97)	167/370	1.72 (0.44,6.73)	1.85 (−0.73,4.43)	0.159
	Primary	380/1009	0.98 (0.25,3.85)	1617/2679	1.65 (0.43,6.43)	1.56 (−1.08,4.19)	0.247
	No education	69/138	1.77 (0.37,8.46)	677/1006	2.00 (0.50,7.92)	1.11 (−2.80,5.03)	0.578

* *p* value for RERI. ORs were adjusted for other covariates. RERI, relative excess risk due to interaction; OR, odds ratio; CI, confidence interval; M, month; Y, year.

## Data Availability

All datasets are available upon request from the DHS website (https://dhsprogram.com/).

## Supplementary Materials

The following are available online at https://www.mdpi.com/article/10.3390/ijerph18105184/s1, Table S1: Prevalence of stunting among under-5 children in the rural and urban areas based on 1991–2016 Tanzania Demographic and Health Surveys.
