# Supplementary material for: Urban–Rural Disparities in the Magnitude and Determinants of Stunting among Children under Five in Tanzania: Based on Tanzania Demographic and Health Surveys 1991–2016
Source: Int J Environ Res Public Health. 2021 May 13;18(10):5184. doi: 10.3390/ijerph18105184 (PMC8153115; doi:10.3390/ijerph18105184)
Supplement: Supplementary file 1 [file ijerph-18-05184-s001.zip › ijerph-1206656-supplementary.pdf]

Supplementary material

Table S1. Prevalence of stunting among under-5 children in the rural and urban areas based on 1991-2016 Tanzania Demographic and Health Surveys.

Region, prevalence % (95%CI)	1991-1992	1996	1999	2004-2005	2009-2010	2015-2016	χ^2 for trend	P
Rural	50.48(49.15,51.80)	52.12(50.71,53.52)	52.66(50.51,54.80)	46.71(45.46,47.97)	44.62(43.31,45.92)	38.26(37.08,39.43)	290.39	<0.001
Urban	46.80(43.98,49.63)	40.79(37.79,43.80)	29.09(24.70,33.47)	35.44(32.89,37.98)	32.11(29.60,34.62)	25.65(23.85,27.45)	167.58	<0.001
Total	49.81(48.61,51.01)	50.13(48.85,51.41)	48.74(46.78,50.70)	44.64(43.51,45.77)	42.21(41.04,43.37)	35.01(34.01,36.01)	481.63	<0.001

CI, confidence interval.
